# Ablation of DGKα facilitates α‐smooth muscle actin expression via the Smad and PKCδ signaling pathways during the acute phase of CCl_4_ ‐induced hepatic injury

**DOI:** 10.1002/2211-5463.13749

**Published:** 2023-12-27

**Authors:** Keiko Seino, Tomoyuki Nakano, Toshiaki Tanaka, Yasukazu Hozumi, Matthew K. Topham, Kaoru Goto, Ken Iseki

**Affiliations:** ^1^ Department of Anatomy and Cell Biology Yamagata University School of Medicine Japan; ^2^ Department of Cell Biology and Morphology Akita University Graduate School of Medicine Japan; ^3^ Huntsman Cancer Institute University of Utah Salt Lake City UT USA; ^4^ Department of Emergency and Critical Care Medicine, School of Medicine Fukushima Medical University Fukushima Japan

**Keywords:** diacylglycerol kinase, hepatic stellate cell, protein kinase C, Smad, α‐smooth muscle actin

## Abstract

Expression of α‐smooth muscle actin (αSMA) is constitutive in vascular smooth muscle cells, but is induced in nonmuscle cells such as hepatic stellate cells (HSCs). HSCs play important roles in both physiological homeostasis and pathological response. HSC activation is characterized by αSMA expression, which is regulated by the TGFβ‐induced Smad pathway. Recently, protein kinase C (PKC) was identified to regulate αSMA expression. Diacylglycerol kinase (DGK) metabolizes a second‐messenger DG, thereby controlling components of DG‐mediated signaling, such as PKC. In the present study we aimed to investigate the putative role of DGKα in αSMA expression. Use of a cellular model indicated that the DGK inhibitor R59949 promotes αSMA expression and PKCδ phosphorylation. It also facilitates Smad2 phosphorylation after 30 min of TGFβ stimulation. Furthermore, immunocytochemical analysis revealed that DGK inhibitor pretreatment without TGFβ stimulation engenders αSMA expression in a granular pattern, whereas DGK inhibitor pretreatment plus TGFβ stimulation significantly induces αSMA incorporation in stress fibers. Through animal model experiments, we observed that DGKα‐knockout mice exhibit increased expression of αSMA in the liver after 48 h of carbon tetrachloride injection, together with enhanced phosphorylation levels of Smad2 and PKCδ. Together, these findings suggest that DGKα negatively regulates αSMA expression by acting on the Smad and PKCδ signaling pathways, which differentially regulate stress fiber incorporation and protein expression of αSMA, respectively.

## Graphical abstract

Expression of α‐smooth muscle actin (αSMA) is a marker for activated nonmuscle cells such as hepatic stellate cells (HSCs). The present study investigated the putative role of diacylglycerol kinase α (DGKα) in αSMA expression. Studies using DGK inhibitor R59949 and DGKα‐KO mice upon CCl_4_‐induced hepatic injury show that DGKα negatively regulates αSMA expression via the PKCδ and Smad2 pathways.

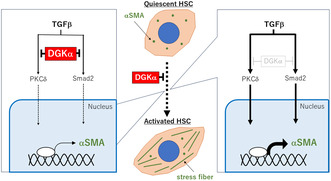

**Abbreviations**
CCl_4_
: carbon tetrachloride
DG: diacylglycerol
DGK: diacylglycerol kinase
GOT: glutamic‐oxaloacetic transaminase
GPT: glutamate‐pyruvate transaminase
HSC: hepatic stellate cells
PKC: protein kinase C
TGFβ: transforming growth factor β
αSMA: α‐smooth muscle actin

The α‐smooth muscle actin (αSMA) is involved in the contractile force of vascular smooth muscle cells. Recent evidence shows that αSMA also plays a critical role in nonmuscle cells such as hepatic stellate cells (HSCs) [1, 2, 3]. HSCs are a resident nonparenchymal liver cell population that serves as a lipid‐storing cell under physiological conditions [3]. Under pathological conditions, however, they proliferate and undergo dramatic phenotypical activation through αSMA. Within HSCs, αSMA is incorporated into stress fibers, which provide an increased myofibroblast contractile force [4], thereby promoting tissue remodeling of the hepatic lobule. Therefore, HSCs are now appreciated as a remarkable plastic cell type that regulates hepatic growth, immunity, inflammation, energy, and nutrient homeostasis under physiological, as well as hepatic fibrosis under pathological conditions [5]. Reportedly, in patients with hepatic fibrosis and experimental models, increased expression of cytokines, especially transforming growth factor β (TGFβ), appears to be a key mediator in the initial step for liver fibrogenesis [6, 7]. Smad signaling plays a major role in the TGFβ pathway, which indicates that the TGFβ‐Smad signaling pathway is a potential target for therapy [8].

Protein kinase C (PKC) isozymes, which belong to a family of serine/threonine kinases, are classified into conventional (α, β, and γ), novel (δ, η, and ε) and atypical subfamilies. Numerous reports describe PKCs as involved in a wide range of biological events, including cell proliferation and differentiation, inflammation, and apoptosis [9, 10]. In an earlier study, an inhibitor of PKC was shown to suppress hepatic fibrosis development effectively [11]. More specifically, TGFβ‐induced αSMA production is reduced significantly by a specific PKCδ inhibitor [12]. These findings suggest that PKC signaling plays another key role in the pathogenesis of hepatic injury. Because PKCδ, one isozyme of novel PKCs, is activated by binding a lipid second‐messenger diacylglycerol (DG), we infer that DG metabolism serves as an additional layer to regulate hepatic injury similarly to Smad signaling.

Diacylglycerol kinase (DGK) is an enzyme that converts DG into phosphatidic acids (PA), thereby regulating two signaling pathways involving these lipid messengers [13, 14, 15, 16]. The present study was conducted to ascertain how DGKs are implicated in the pathogenesis of liver injury. We specifically examined the regulation of αSMA expression by PKC and Smad signaling pathways. To this end, we used a cell culture model experiment using the DGK inhibitor R59949. Additionally, we performed an animal model experiment using carbon tetrachloride (CCl_4_), a chemical that induces hepatocyte necrosis and αSMA expression in activated HSCs at an acute phase [17, 18].

The results of an animal model of CCl_4_‐induced hepatic injury show that DGKα ablation enhances αSMA expression coinciding with increased Smad2 and PKCδ phosphorylation, suggesting that DGKα exerts a negative regulation on αSMA expression. We also evaluated the extent of CCl_4_‐induced hepatic injury and discuss the functional implication of αSMA expression in HSCs of DGKα‐KO mice after CCl_4_ injury.

## Materials and methods

### Cell culture

Mouse fibroblast cell line NIH3T3 cells were obtained from RIKEN BRC (Tsukuba, Japan) and were cultured in Dulbecco's modified Eagle medium (DMEM) supplemented with 10% fetal bovine serum and Penicillin G / Streptomycin under 5% CO_2_ at 37 °C. Cells were incubated with transforming growth factor β (TGFβ) (Millipore Sigma, Burlington, MA, USA) at 10 ng·mL^−1^ or dimethyl sulfoxide (DMSO) as a vehicle. In some experiments, cells were pretreated with DGK inhibitor R59949 (Millipore Sigma) at 10 μm. Cells were harvested at 0, 30 min, and 6 h of incubation after TGFβ treatment for immunoblot analysis.

### Immunocytochemistry

For immunocytochemical analysis, cells were fixed in 2% paraformaldehyde / 0.1 m phosphate buffer (pH 7.4). After fixation, cells were perforated with 0.3% Triton‐X 100 / phosphate buffered saline (PBS), followed by blocking the nonspecific binding site using 5% normal goat serum (NGS)/PBS, and were reacted with anti‐α‐smooth muscle actin (1:100; Millipore Sigma). Immunoreaction was visualized by anti‐rabbit IgG‐Alexa 488 (Thermo Fisher Scientific, Waltham, MA, USA). Nuclear staining was performed with DAPI. Confocal microscopic observation was carried out using LSM700 (Carl Zeiss, Jena, Germany).

### Animal models

All animal experiments were carried out in accordance with the guidelines and permission of the Yamagata University Animal Ethics Committee (approval number: R5012). Mice were housed under standard conditions and maintained on a 12‐h light/dark cycle. They had free access to water and were fed standard mouse laboratory chow (F‐2; Oriental Yeast Co., Tokyo, Japan). An acute mouse carbon tetrachloride (CCl_4_) model using male 6‐week‐old C57BL6J wildtype (WT) and DGKα‐knockout (KO) mice [19] were established by intraperitoneal injection of CCl_4_ at a dose of 0.6 mL·kg^−1^. Corn oil was injected at an equal amount as a control. After 48 h of injection, mice were sacrificed and the livers were removed for histological and immunoblot analyses under deep anesthesia.

### Immunoblot analysis

Cell lysate and liver samples were homogenized in a buffer containing 20 mm Tris–HCl, 150 mm NaCl, 1 mm EGTA, 1 mm EDTA, 1% Triton X‐100, 2.5% sodium pyrophosphate, 1 mm β‐glycerophosphate, 1 mm sodium vanadate, 1 mm PMSF, and a complete proteinase inhibitor cocktail (Roche Applied Science, Penzberg, Germany), followed by centrifugation at 12,000 ***g***, for 10 min at 4 °C. Supernatant was used as a protein sample. Protein concentration was determined by BCA protein assay reagent (Thermo Fisher Scientific). Each protein (10 μg) was separated on 10% SDS/PAGE, and transferred to a PVDF membrane. The membranes were incubated in 5% nonfat skim milk/TBS containing 0.05% Tween‐20 to block nonspecific binding of the antibody. The antibodies used in the present study were: αSMA (1:1000; Millipore Sigma), DGKα (1:1000; Proteintech, Rosemont, IL, USA), phospho‐PKCδ (Thr505) (1:1000; Cell Signaling Technology, Beverly, MA, USA), total PKCδ (1:1000; Cell Signaling Technology), vimentin (1:1000; Abcam, Cambridge, UK), phospho‐Smad2 (1:1000; Cell Signaling Technology), toral Smad2/3 (1:1000; Cell Signaling Technology), β‐actin (1:4000; Cell Signaling Technology) and GAPDH (1:1000; Cell Signaling Technology). Immunoreactive bands were visualized by using Immobilon Western (Millipore Sigma). Band intensities were quantified by densitometry using image j (National Institutes of Health, Bethesda, MD, USA) as described [20].

### Histological analysis

Livers removed from CCl_4_‐induced models and controls were fixed in 4% paraformaldehyde / 0.1 m phosphate buffer at 4 °C overnight. Paraffin and frozen sections were cut into 6‐ and 20‐μm thickness, respectively. Frozen sections were used for αSMA‐immunohistochemistry and paraffin sections were used for hematoxylin and eosin (H&E) and DGKα‐immunofluorescence.

### αSMA immunohistochemistry

Frozen sections were soaked in 0.3% Triton X‐100/PBS at room temperature (RT) for 30 min. Endogenous peroxidase was inactivated by 0.3% H_2_O_2_. After blocking with 5% NGS/PBS, sections were incubated with anti‐αSMA antibody (1:400; Millipore Sigma) in a moist chamber at RT overnight. Immunoreaction was detected with diaminobenzidine tetrachloride.

### DGKα immunofluorescence

Rehydrated paraffin sections were treated with antigen retrieval reagent (HistoVT one, Nacalai Tesque, Kyoto, Japan). Sections were soaked in 0.3% Triton X‐100/PBS at RT for 30 min. After blocking with 5% NGS/PBS, sections were incubated with anti‐DGKα antibody (1:100; Proteintech) in a moist chamber at RT overnight. Immunoreaction was detected with anti‐rabbit IgG‐Alexa488 (Thermo Fisher Scientific). Nuclear staining was performed with DAPI. Confocal microscopic observation was carried out using LSM700 (Carl Zeiss).

### Glutamic‐oxaloacetic transaminase (GOT) and glutamate‐pyruvate transaminase (GPT) assay

Serum GOT/GTP levels were measured according to the manufacturer's instruction using transaminase CII test Wako (Wako Pure Chemical Industries, Osaka, Japan).

### TGFβ assay

Serum TGFβ levels were measured according to the manufacturer's instruction using the Quantikine ELISA TGFβ1‐immunoassay kit (R&D Systems, Minneapolis, MN, USA).

## Results

### Cell culture model using DGK inhibitor

**Fig. 1:**
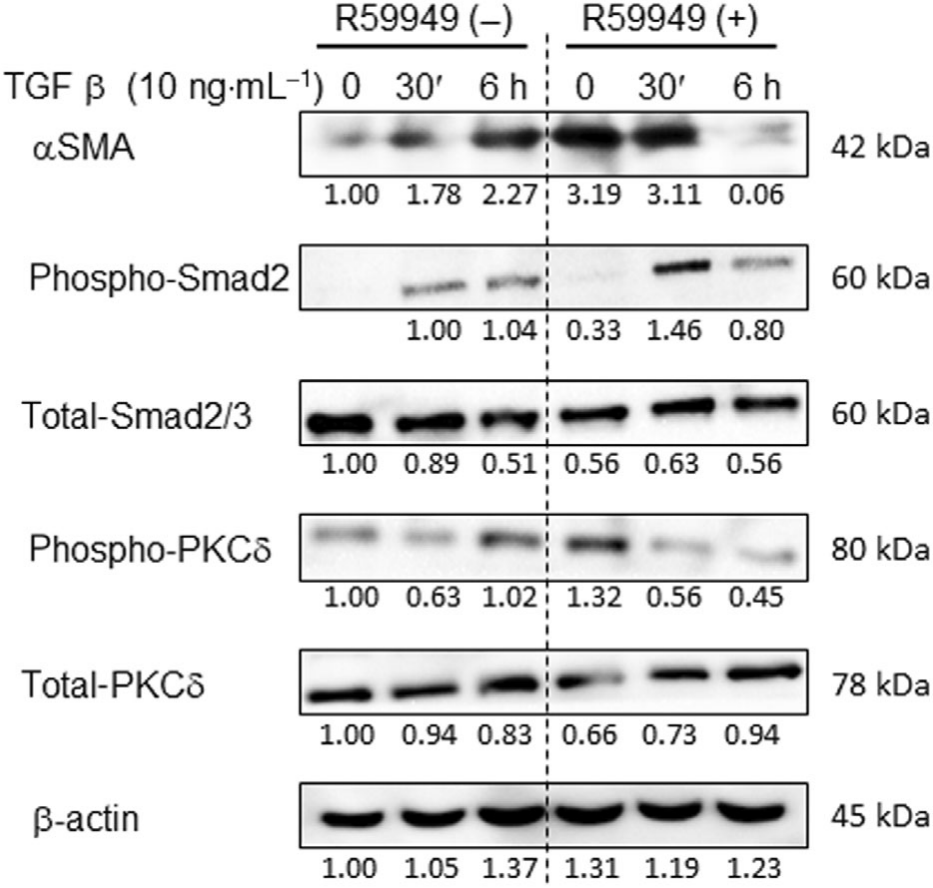
Immunoblot analysis in cell culture model using DGK inhibitor. Nontransformed NIH3T3 cells derived from mouse fibroblasts were incubated with TGFβ (10 ng·mL^−1^). Some cells were pretreated for 1 h with R59949, an inhibitor for class I DGKs (10 μm). Control cells were incubated with DMSO solvent alone. Cells were harvested at 0, 30 min, and 6 h after TGFβ stimulation in the presence or absence of R59949. Total cell lysates were immunoblotted for αSMA, phospho‐PKCδ (Thr505), total PKCδ, phospho‐Smad2, and total Smad2/3. β‐actin was used as a control. Immunoblot signals were quantified by densitometry and the values were normalized to the β‐actin level. A representative result of three repeated experiments is shown.

We first examined the effect of DGK inhibitor R59949 on αSMA expression and signaling pathway at the cellular level. We used the untransformed mouse fibroblast cell line NIH3T3 cells. Reportedly, a broad DGK inhibitor R59949 inhibits the enzymatic activity of class I DGK isozymes including DGKα, DGKβ, and DGKγ [21]. Since an earlier study shows that major DGK isozymes expressed in NIH3T3 cells include DGKα, DGKδ, and DGKζ [22], R59949 is presumed to inhibit DGKα activity mostly, if not entirely, in this cell line. Earlier studies have reported that αSMA expression is positively regulated by TGFβ that is secreted by hepatocytes and resident macrophages designated as Kupffer cells [23]. As shown in Fig. 1, immunoblot analysis revealed that in the absence of R59949, αSMA expression is increased at 30 min and 6 h in response to TGFβ stimulation. When pretreated with R59949 for 1 h before stimulation, αSMA expression was increased significantly at timepoint 0 (without TGFβ stimulation). It remained high at 30 min. However, it had retuned toward the baseline when measured 6 h after TGFβ stimulation.

Reportedly, Smad2 and PKCδ play roles in TGFβ‐induced αSMA expression. Therefore, we next investigated the regulatory mechanism of αSMA expression using a DGK inhibitor. As shown in Fig. 1, the results showed that, in the absence of R59949, Smad2 activity as assessed by phosphorylation status is increased at 30 min and 6 h after TGFβ stimulation. In the presence of R59949, Smad2 activity was significantly higher at 30 min of stimulation compared with the control without R59949. Regarding PKCδ, in the absence of R59949, its phosphorylation status was increased at 6 h after TGFβ stimulation. However, R59949 pretreatment alone increased significantly the PKCδ phosphorylation levels at timepoint 0. They were decreased thereafter. In this regard, PKCα/β phosphorylation levels were not changed during the course of TGFβ stimulation with or without R59949 (data not shown).

The results of the immunoblot analysis can be summarized as follows. Under conditions of DGK inhibitor R59949 pretreatment, αSMA expression and PKCδ activity are upregulated without TGFβ stimulation. On the other hand, in the presence of R59949, Smad2 activity is upregulated after 30 min of TGFβ stimulation. These findings suggest that class I DGK activity inhibition acts differently on two signaling pathways. It should be also noted, however, that αSMA expression declined in R59949‐pretreated cells after 6 h of TGFβ stimulation. The reason for this remains undetermined, although a negative feedback loop might serve to restrain αSMA expression at 6 h.

**Fig. 2:**
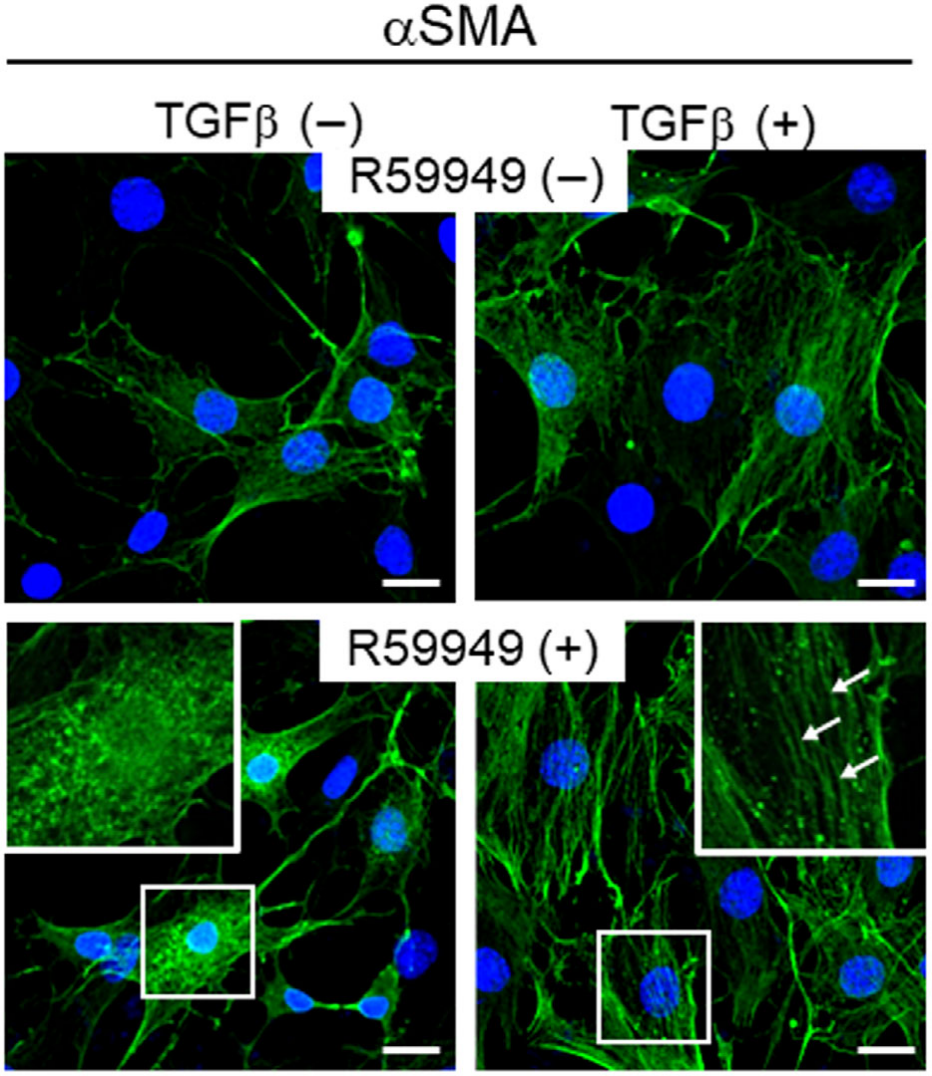
Immunocytochemical analysis in cell culture model using DGK inhibitor. NIH3T3 cells were stimulated with TGFβ in the presence or absence of R59949. After 30 min, cells were fixed and stained for αSMA (green). In the presence of R59949, αSMA immunoreactivity was significantly increased before and after TGFβ stimulation. Note that αSMA staining is detected in a granular pattern before TGFβ stimulation whereas it is observed in a stress fiber pattern (arrows) after TGFβ stimulation. Insets are the magnified images of the squares. Scale bars = 10 μm.

TGFβ stimulates αSMA synthesis and incorporation into stress fibers [4]. Stable incorporation of αSMA into stress fibers provides an increased myofibroblast contractile force that participates in tissue remodeling [4]. Therefore, we next performed immunocytochemistry to examine the morphological aspects of αSMA using the same experimental protocol (Fig. 2). In the absence of R59949 (upper panels), αSMA labeling was observed faintly in a fine granular pattern without TGFβ stimulation. After 30 min of TGFβ stimulation, αSMA was effectively incorporated partially into stress fibers (upper right panel).

When pretreated with R59949 for 1 h before stimulation (Fig. 2, lower panels), αSMA labeling was observed as abundant in a punctate, cytoplasmic granular pattern, although no apparent stress fiber formation was recognized (lower left panel). At 30 min of TGFβ stimulation, most of the αSMA labeling was obviously visible in a cytoskeletal pattern, suggesting that αSMA is incorporated efficiently into stress fibers (lower right panel). Together, the results suggest that the class I DGK inhibitor R59949 enhances αSMA protein expression irrespective of TGFβ stimulation, and that it facilitates αSMA incorporation into stress fibers in response to TGFβ stimulation.

### Hepatic injury model

Reportedly, DGKα is intimately involved in liver function under pathophysiological conditions [24] and positively regulates proliferation and invasion of human hepatocellular carcinoma cells [25]. Immunohistochemical analysis showed that DGKα immunoreactivity is diffusely detected throughout the hepatic lobule. Importantly, it was clearly visible in cells with a slender shape and long cytoplasmic extensions, suggesting that DGKα is expressed in HSCs, if not solely (arrows in upper left panel of Fig. 5A). Therefore, we focused on DGKα in an animal model and strove to gain further insight into the functional implications of DGKα in αSMA expression in HSCs. We undertook the CCl_4_ intoxication model, which engenders αSMA induction. In addition to DGKα‐KO, we used DGKε‐KO and DGKζ‐KO mice as controls.

Activation of HSCs is a well‐known event, occurring at the beginning of CCl_4_‐induced hepatic injury. Reportedly, a single dose of CCl_4_ (0.6 mL·kg^−1^ body weight) is sufficient to elicit liver damage as quickly as 24 h after injection [26]. In fact, αSMA, a widely characterized cytoskeletal protein, represents the hallmark of myofibroblast activation and differentiation in liver injury. Therefore, we next examined the levels of αSMA as a marker for HSC activation in an acute injury model.

**Fig. 3:**
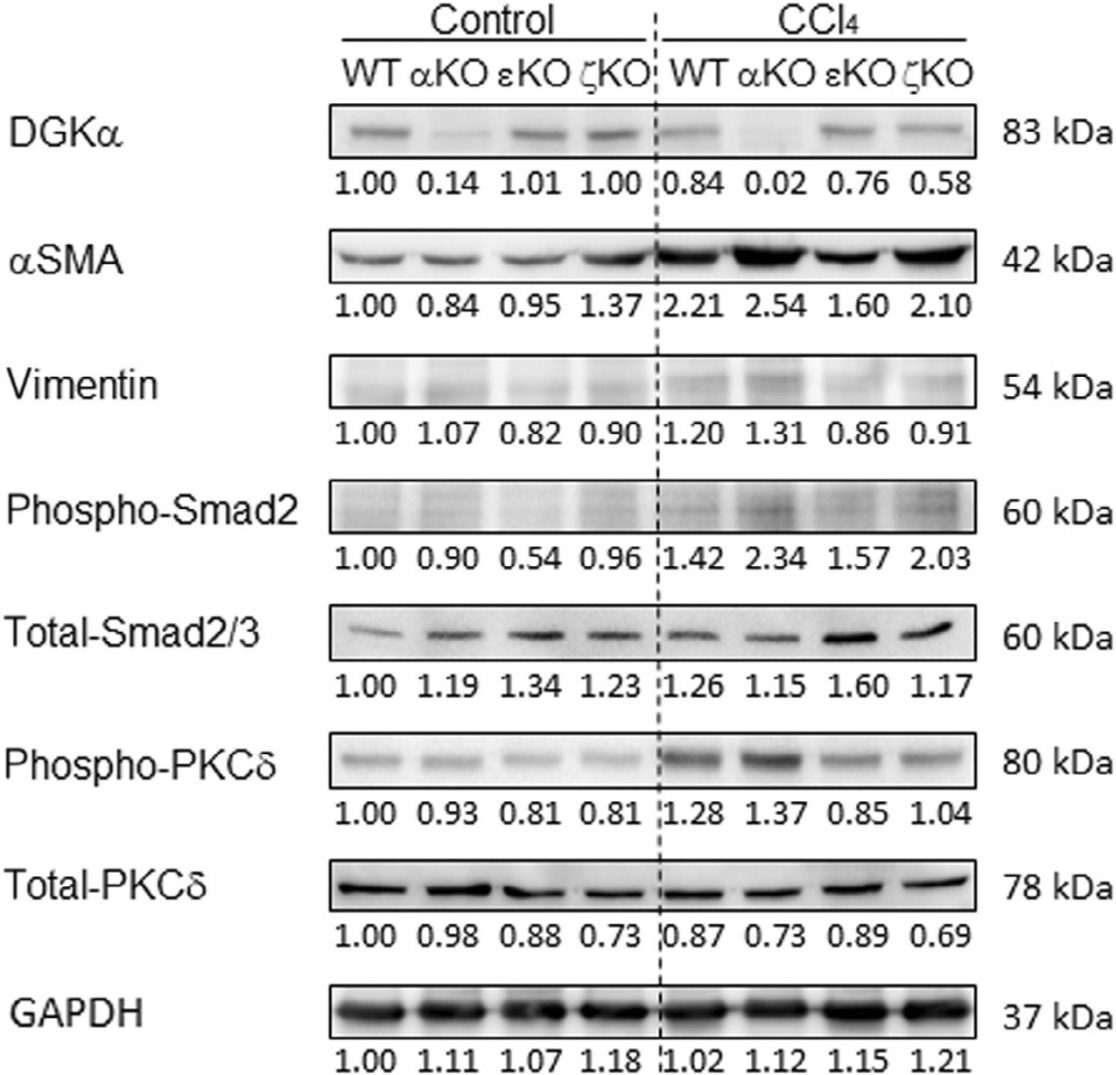
Immunoblot analysis in acute hepatic injury model. Wildtype (WT) and DGKα‐knockout (DGKα‐KO) mice were injected with a single dose of CCl_4_ (0.6 mL·kg^−1^ body weight) or corn oil (control). After 48 h of injection, livers were removed and examined for immunoblot analyses. As a control, corn oil was injected with the same amount. DGKε‐KO and DGKζ‐KO mice were used as controls. Liver homogenates were immunoblotted for αSMA, phospho‐PKCδ (Thr505), total PKCδ, vimentin, phospho‐Smad2, and total Smad2/3. GAPDH was used as a control. Immunoblot signals were quantified by densitometry and the values were normalized to the GAPDH level. A representative result of three repeated experiments is shown.

In immunoblot analysis, it should be noted first that in WT liver after 48 h of CCl_4_ injection, DGKα protein expression levels are decreased, whereas αSMA levels are significantly increased (Fig. 3). In this regard, the αSMA levels were robustly increased in DGKα‐deficient liver. Similar results were obtained in the expression levels of vimentin, another HSC activation marker. These results suggest that decreased DGKα levels lead to upregulated HSC activation upon CCl_4_ intoxication. We next examined the activation status of upstream pathways regulating αSMA expression. Upon CCl_4_ injection, the phosphorylation levels of Smad2 and PKCδ were significantly increased in WT liver that exhibited decreased DGKα expression levels. Consistent with this, phosphorylation levels of those proteins were vigorously enhanced in DGKα‐deficient liver.

In DGKε‐ and DGKζ‐deficient livers, DGKα protein levels were decreased, and αSMA expression levels were significantly increased upon CCl_4_ injection, showing an inverse relationship between levels of DGKα expression and αSMA induction. This is principally consistent with the results obtained in WT and DGKα‐KO mice. However, the details seemed somewhat different: Levels of PKCδ phosphorylation were not significantly increased in DGKε‐ and DGKζ‐KO mice. The absence of DGKε or DGKζ might exert some yet‐unknown effects on the response to CCl_4_ intoxication.

**Fig. 4:**
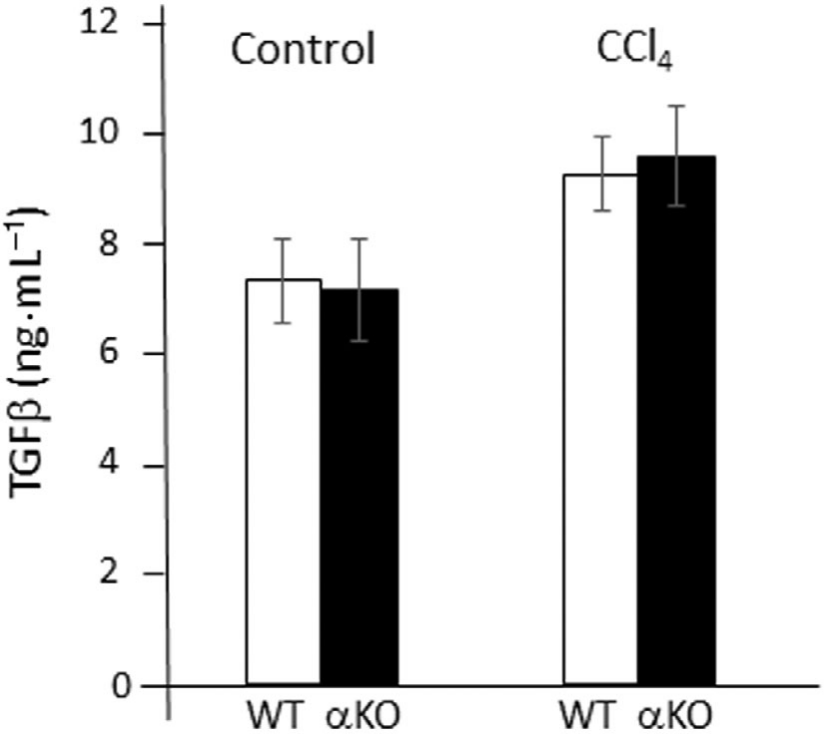
Measurement of the serum TGFβ levels for liver injury. TGFβ levels were measured using the Quantikine ELISA TGFβ‐immunoassay kit. Serum TGFβ levels were increased slightly in both WT and DGKα‐KO mice to the same extent 48 h after CCl_4_ injection. Data shown are the means ± SD (*n* = 5). n.s., not significant (Student's *t*‐test).

We next examined whether TGFβ secretion levels are altered in DGKα‐KO mice 48 h after CCl_4_ injection. To this end, we performed an enzyme‐linked immunosorvent assay (ELISA) for serum TGFβ levels. As presented in Fig. 4, serum TGFβ levels were increased slightly in both WT and DGKα‐KO mice to the same extent 48 h after CCl_4_ injection. The results suggest that increased αSMA expression is not attributed to an increased TGFβ secretion, but rather to the activated signaling pathway downstream of the TGFβ receptor in HSCs of DGKα‐KO mice.

**Fig. 5:**
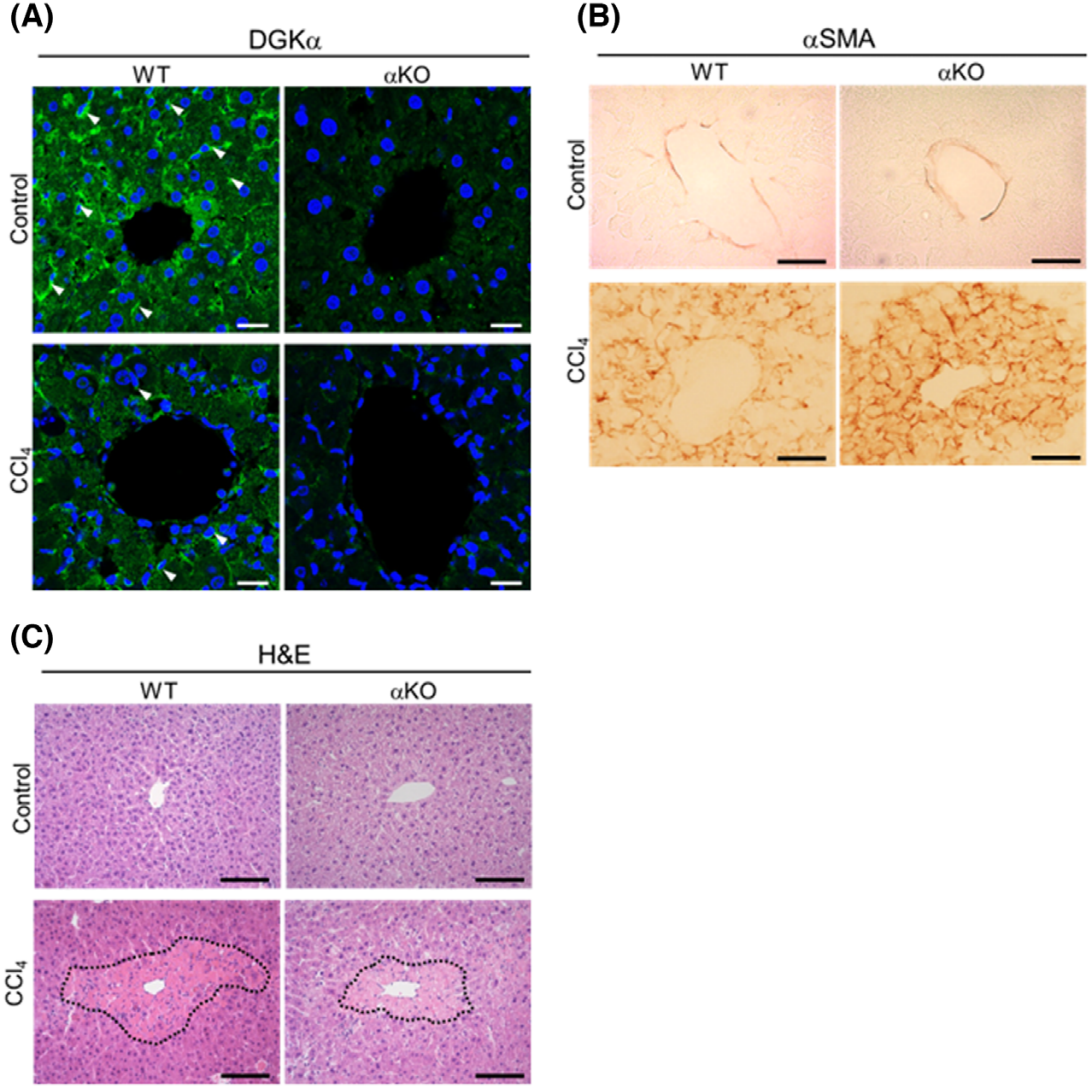
Histological analysis of the livers in the acute hepatic injury model of WT and DGKα‐KO mice. (A) Immunofluorescence using the anti‐DGKα‐antibody. Compared with the corn oil‐injected control (upper panel), smaller numbers of DGKα‐immunoreactive cells (arrowheads) are demonstrated in CCl_4_‐injected WT liver (lower panel). No immunoreaction for DGKα was detected in DGKα‐KO liver. Scale bar = 10 μm. (B) αSMA‐immunoreactivity is intensely recognized in CCl_4_‐injected DGKα‐KO liver compared with WT liver (lower panels). Upper panels are oil‐injected controls. Scale bar = 50 μm. (C) Paraffin sections of livers were stained with routine H&E. Note the smaller necrotic area indicated by a dashed line in DGKα‐KO mice compared with WT controls. Scale bar = 50 μm.

Having shown that upon CCl_4_ intoxication DGKα‐deficient livers exhibit activated signaling pathways of Smad2 and PKCδ that lead to enhanced αSMA expression, we next assessed how these conditions affect the progress or degree of hepatic damage after 48 h of CCl_4_ injection in DGKα‐KO mice. The acute liver injury model of this experimental scheme is shown to induce centrilobular necrosis, which is characterized by hepatocyte necrosis and lymphocyte infiltration around a central vein [17]. As shown in Fig. 5A, confocal microscopic observation demonstrated that the number of DGKα immunoreactive cells was decreased in CCl_4_‐injected WT liver compared with that of oil‐injected WT liver. These data were consistent with the immunoblot analysis. The results also revealed that after 48 h of CCl_4_ injection, αSMA‐immunoreactive cells was observed in the centrilobular portion of the liver in both WT and DGKα‐KO mice (Fig. 5B). Consistent with the immunoblot analysis, αSMA‐immunoreactive cells were significantly more abundant in DGKα‐deficient livers than in WT ones. We next performed H&E staining to examine the degree of hepatic damage after 48 h of CCl_4_ injection. On liver sections of both WT and DGKα‐KO mice, hepatocytes exhibited a paler cytoplasm together with pyknotic nuclei in the centrilobular area, showing centrilobular hepatic damage at an acute phase (Fig. 5C). It should be noted, however, that the necrotic area seems slightly smaller in DGKα‐deficient liver than in WT liver.

**Fig. 6:**
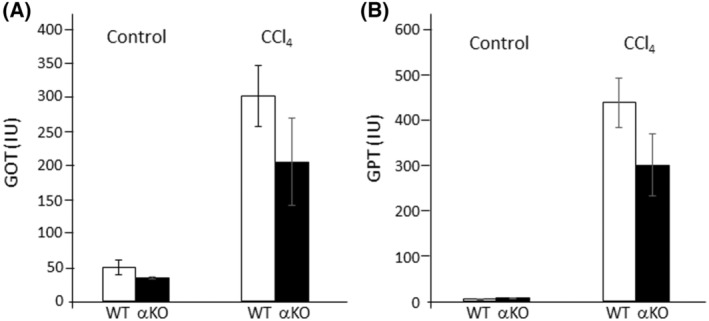
Measurement of the serum markers for liver injury. Serum GOT (A) and GPT (B) levels were measured using the transaminase CII test, Wako. Both levels tend to be lower in DGKα‐KO mice than WT controls, although no significant difference was discerned. Data shown are the means ± SD (*n* = 4–7). n.s., not significant (Student's *t*‐test).

Serum glutamic‐oxaloacetic transaminase (GOT) / glutamate‐pyruvate transaminase (GPT) assays are useful biochemical markers to assess hepatocyte damage. To be noted, after 48 h of CCl_4_ injection GOT and GPT levels tended to be lower in DGKα‐KO mice than those in WT mice, although statistically not significant (Fig. 6). Collectively, the results suggest that DGKα‐deficient liver is less vulnerable to CCl_4_‐induced injury than WT liver, judging from the smaller centrilobular necrotic area and lower serum biomarkers, despite the upregulated αSMA expression.

## Discussion

αSMA, a cytoskeletal protein in vascular smooth muscle cells, is induced in HSCs under pathological conditions. This represents an initial step for HSC activation [2]. In the present study we examined the functional implication of DGK in the regulatory mechanism of αSMA expression.

First, we reveal that in a cellular model experiment, DGK inhibitor R59949 pretreatment enhances αSMA expression in NIH3T3 cells. Previous studies showed that DGKα, DGKδ, and DGKζ are major DGK isozymes expressed in NIH3T3 cells [22] and that DGK inhibitor R59949 mainly acts on class I DGKs (DGKα, DGKβ, and DGKγ) [21]. Therefore, it is reasonable to consider DGKα as a major target of R59949 in NIH3T3 cells. Taken these findings together, we assumed that enhanced αSMA induction is attributed to inhibition of DGKα enzymatic activity in NIH3T3 cells, if not entirely.

This assumption is confirmed by our second experiment, i.e. an animal model of acute hepatic injury. In the liver of WT mice, CCl_4_ intoxication downregulates DGKα expression levels but upregulates αSMA induction. Consistent with this, αSMA induction is robustly upregulated in the liver of DGKα‐KO mice upon CCl_4_ intoxication. Collectively, the results obtained from studies using cellular and animal models suggest that DGKα negatively regulates αSMA expression in an activity‐dependent manner.

In CCl_4_‐induced liver injury, TGFβ appears to be a key mediator [6, 7]. The TGFβ–activated Smad signaling pathway stimulates experimental hepatic fibrosis and is a potential target for therapy [8]. In this regard, we found no significant difference in serum TGFβ levels between wildtype and DGKα‐KO mice in an acute hepatic injury model.

From these findings, we infer that increased αSMA expression is assigned to a facilitated response in HSCs themselves. As described above, the TGFβ‐activated Smad signaling pathway plays a pivotal role in HSC activation. Our cellular model study suggests that inhibition of DGKα enzymatic activity enhances Smad2 phosphorylation together with αSMA expression at 30 min of TGFβ stimulation. Additionally, it is noteworthy that DGKα activity inhibition enhances PKCδ phosphorylation together with αSMA expression in the absence of TGFβ stimulation. When considering the time course of activation of Smad2 and PKCδ together with that of subcellular αSMA labeling, we propose the following working hypothesis: DGKα activity inhibition enhances αSMA protein synthesis in HSCs independently of TGFβ stimulation, but it does not facilitate stress fiber formation. In response to TGFβ stimulation, DGKα activity inhibition enhances Smad2 activation, which engenders the effective incorporation of αSMA into stress fibers.

It remains unclear why αSMA expression declines in R59949‐pretreated cells after 6 h of TGFβ stimulation. Since cytoskeletal proteins like α‐SMA should be tightly regulated within the physiological range for cell survival, a yet undetermined negative feedback loop might serve to restrain αSMA expression at 6 h.

An important question remains unanswered, which involves the phenotype of DGKα‐deficient liver. In the animal model of the present study, we found that DGKα‐deficient liver exhibits a smaller necrotic area together with lower GOT/GPT levels than WT liver. This suggests that DGKα‐deficiency renders cells less vulnerable to CCl_4_‐induced injury compared with WT liver, despite the upregulated αSMA expression.

Does DGKα ablation induce HSC activation, thereby ameliorating liver injury? As noted in the Introduction, recent studies suggest that HSCs represent a remarkable plastic cell type that regulates both liver homeostasis and tissue repair [5, 27]. Whether HSC activation initiates repair processes or exacerbates liver damage might depend on the cellular and environmental context and the extent of injury. Further studies are needed to elucidate this point.

In summary, the present studies using cellular and animal models show that DGKα activity inhibition and DGKα ablation leads to activation of PKCδ and TGFβ‐triggered Smad2 pathways, thereby cooperatively inducing αSMA expression for HSC activation. These findings suggest that DGKα negatively regulates αSMA expression, which may be exerted in an activity‐dependent manner. In addition, we also suggest that the PKCδ pathway regulates αSMA expression levels, whereas the TGFβ‐triggered Smad pathway facilitates αSMA incorporation into stress fibers. Interestingly, it is also suggested that DGKα ablation does not result in exacerbation of liver damage in CCl_4_ injection, despite the upregulated αSMA expression. This raises a possibility that HSC activation is involved in the recovery process in the DGKα‐mediated pathway.

## Conflict of interest

Kaoru Goto received research funding from Ono Pharmaceutical Co., Ltd. The other authors have no conflicts of interest.

### Peer review

The peer review history for this article is available at https://www.webofscience.com/api/gateway/wos/peer‐review/10.1002/2211‐5463.13749.

## Author contributions

KS designed the study and performed the experiments. TN designed the study, performed the experiments, and wrote the article. TT and YH performed the experiments. MKT designed and supervised the study. KG designed the study and wrote the article. KI conceived and supervised the study.

## Data Availability

All data and materials are available upon reasonable request. Address to T.N. (e‐mail: t-nakano@med.id.yamagata-u.ac.jp). Department of Anatomy and Cell Biology, Yamagata University School of Medicine, Yamagata, Japan.
